# A hybrid CNN-Transformer network integrating multiscale spatially detailed features for medical image segmentation

**DOI:** 10.1371/journal.pone.0345549

**Published:** 2026-04-29

**Authors:** Bo Li, Wei Zhou, Haijun Li

**Affiliations:** 1 School of Computer and Software Engineering, Xihua University, Chengdu, Sichuan, China; 2 Department of Cancer Center, The Second People’s Hospital of Neijiang, Neijiang, Sichuan, China; Samsun University: Samsun Universitesi, TÜRKIYE

## Abstract

The rapid advancement of deep learning has established Convolutional Neural Networks (CNNs) as mainstream for medical image segmentation, yet their limited receptive field hinders long-range dependency capture. While Transformers excel at modeling global features via self-attention, their high computational complexity burdens high-resolution image processing. To leverage the complementary strengths of both architectures and integrate local and global features under a lightweight framework for enhanced accuracy and efficiency, this work proposes a novel encoder based on parallel CNN and Swin Transformer. Its effective integration is the Semantics and Detail Infusion (SDI) module, which fuses multi-scale features and employs attention to prioritize critical details, enriching features for decoder resolution recovery. Evaluations were conducted on two publicly available datasets, namely the Synapse Multi-Organ Segmentation dataset and the Aortic Vessel Tree dataset. The proposed model achieved Dice coefficients of 84.19% and 87.91%, respectively, and corresponding Hausdorff Distances of 12.64 mm and 7.06 mm. These results represent significant improvements over the UNet benchmark, with Dice score gains of 7.34% and 5.02%, respectively. The results further underscore the model’s robustness, efficiency, and clinical relevance in accurately delineating complex anatomical structures, particularly in abdominal segmentation tasks. By effectively fusing CNN and Transformer advantages, our approach meets high-performance standards for medical image segmentation while offering practical benefits for real-world clinical deployment in resource-constrained environments. The code is publicly available on https://github.com/Palpitate-v/HybridNet.

## Introduction

Medical image segmentation techniques have increasingly played a pivotal role in aiding clinical diagnosis [1]. Precisely and robustly delineating lesion regions within diverse and complex image backgrounds has thus become a major research focus. Traditional segmentation methods—such as region-based, edge-based, and thresholding approaches [2]—depend on handcrafted features and suffer from limited generalization. Deep learning, by contrast, can be used to extract information from very complex images [3]. With the rise of deep learning, in particular fully convolutional neural networks (FCNNs) [4] and convolutional neural networks (CNNs) [5], exemplified by UNet [6] and its variants [7], the use of encoder and decoder architectures together with skip connections for multiple scale feature fusion has propelled the field forward. However, the inherently limited receptive field of CNNs[5] constrains their ability to capture long distance dependencies, which poses challenges when segmenting lesions with complex textures and blurred boundaries and may increase the risk of clinical misinterpretation.

On the other hand, the success of Transformer models in natural language processing (NLP) [8] has inspired their application to vision tasks. The Vision Transformer (ViT) [9] uses self attention to model global context and address long distance dependency issues.For example, Cell-TRACTR [10], based on a Transformer architecture, demonstrates the effectiveness of self-attention mechanisms in handling complex biological image scenarios. However, pure Transformer approaches face two major challenges: difficulty in extracting fine grained low level features [11] and quadratic growth of computational cost for self attention as input size increases, which becomes a bottleneck for high resolution three-dimensional medical image processing [12]. To reduce the computational burden, the Swin Transformer [13] introduces a hierarchical architecture together with a shifted window mechanism. Based entirely on this design, SwinUnet [14] achieves a significant reduction in computation, but in abandoning all convolutional operations it fails to exploit the strength of convolution in local feature extraction.

Consequently, researchers have turned to hybrid architectures that combine the strengths of CNNs and Transformers. For example, TransUNet [15] employs a two branch structure to balance global context modeling and local feature extraction, improving segmentation accuracy at the expense of added complexity. Its key limitation lies in its skip connections, which simply concatenate feature maps without accounting for semantic differences between levels and thus undermine effective multi scale feature fusion. ParaTransCNN [16] incorporates squeeze and excitation modules [17] to adaptively recalibrate channel features in its skip connections, but it still lacks an explicit spatial attention mechanism to preserve structural continuity, limiting its ability to delineate irregular lesion boundaries. U-Net v2 [18] enhances boundary precision within a pure CNN framework by adding a semantic and detail integration module that fuses multiple level feature maps and refines them via CBAM attention [19], yet it cannot overcome the fundamental receptive field limitation inherent to CNNs. It is also noteworthy that in parallel CNN-Transformer architectures, direct fusion of feature maps from these two branches often leads to semantic inconsistency, noise accumulation and redundant information, all of which severely limit further gains in segmentation accuracy [20].

In summary, existing methods face four core challenges in achieving efficient and accurate multi-scale feature fusion. First, the computational overhead of pure Transformer models or complex hybrid architectures (such as ParaTransCNN [16]) limits their deployment in resource-constrained settings. Second, current skip connection fusion strategies struggle to bridge the semantic gap across hierarchical levels while precisely integrating spatial details. Third, in parallel hybrid architectures, the direct fusion of feature maps from CNN and Transformer branches often results in semantic inconsistency, noise accumulation and redundant information, which hinders effective information utilization. Fourth, the limited and heterogeneous nature of medical imaging data renders models prone to overfitting, thereby compromising final segmentation accuracy and clinical robustness.

To this end, we propose a novel parallel encoder and decoder framework that incorporates the Swin Transformer layered window mechanism to preserve long-range dependency modeling while substantially reducing computational complexity, thereby directly addressing the first challenge. To mitigate insufficient cross-level fusion and redundant or inconsistent branch fusion, we introduce an enhanced semantic-detail integration (SDI) module [18] within the skip connections. This module combines channel attention and spatial attention from CBAM [19] to address the absence of spatial attention in ParaTransCNN and to strengthen the selection of salient features. By optimizing the fusion of feature maps from different encoding levels, the SDI module overcomes the simplistic fusion strategy of TransUNet [15] and significantly enhances the representation of diagnostically critical patterns, in particular boundary details. Considering the limited nature of medical imaging data and the complexity of the model, ablation studies demonstrate that applying Dropout after the computationally intensive Swin Transformer modules and before the final prediction effectively prevents overfitting and substantially improves both segmentation accuracy and generalization.

The main contributions of this paper were:

- We design a parallel encoder-decoder architecture combining CNN and Swin Transformer. The Swin Transformer’s hierarchical structure and windowing mechanism effectively reduce the computational complexity, addressing the quadratic complexity challenge inherent in other Transformer-based methods.
- In this work, we incorporate an effective multi-scale feature fusion technique via the SDI module, which enhances the feature representation in skip connections by integrating channel and spatial attention mechanisms derived from the CBAM framework, thus enabling more effective multi-scale contextual information integration.
- Through extensive ablation experiments, we demonstrate that applying Dropout regularization after the Swin Transformer and before the final prediction prevents overfitting and significantly improves segmentation accuracy.
- We conducted extensive experiments on two publicly available medical image segmentation datasets. The experimental results demonstrate that our proposed architecture outperforms multiple baseline models based on CNNs and Transformers in terms of segmentation performance.

## Related work

### CNNs in medical segmentation

Convolutional neural networks (CNNs) have been receiving much research attention in the field of medical image segmentation. UNet [6] has become an important cornerstone in the field of medical image segmentation since it was first proposed by Ronneberger et al. UNet is well known for its unique encoder-decoder structure and skip connection mechanism, effectively integrating contextual and localization information in images. Continuing research has led scholars to make a series of improvements to UNet, aiming to enhance the segmentation accuracy of the model. For instance, Unet++ [7] adds dense skip connections on top of UNet, significantly enhancing feature transfer and fusion capabilities. ResNet [21] introduces a residual structure, addressing the issues of gradient vanishing or explosion, and enabling the construction of deeper networks. Attention U-Net [22] and Non-local U-Net [23] incorporate attention mechanisms, respectively enhancing the model’s focus on regions of interest. Furthermore, the recent U-Net v2 [18] introduces the SDI (Semantics and Detail Infusion) module, which further enhances the fusion of feature maps by improving skip connections, significantly improving segmentation accuracy and robustness. These advancements have significantly progressed CNN-based medical image segmentation techniques, improving accuracy, robustness, and computational efficiency.

### Vision transformers in medical segmentation

In 2020, the Google team first proposed the Vision Transformer (ViT) model for image recognition [9]. The model utilizes the coding structure of the Transformer to generate contextual features by computing pairwise interactions between image patches, thus effectively addressing long-range dependencies. Despite its relatively simple architecture, ViT demonstrates excellent performance and strong scalability. Experiments demonstrate that ViT outperforms traditional Convolutional Neural Networks (CNNs) when sufficient data is available for pre-training [24]. Although ViT shows excellent performance on large-scale datasets, its computational complexity is high when processing high-resolution images. To address this issue, Liu et al. [13] proposed the Swin Transformer, introducing a window-based multi-head self-attention (W-MSA) module and a shifted window-based multi-head self-attention (SW-MSA) module. These mechanisms divide the input image into non-overlapping windows and the self-attention computation is limited to the elements within each window, thus avoiding the quadratic complexity of the traditional Transformer. This avoids the quadratic complexity of the global self-attention computation in the traditional Transformer and reduces it to linear complexity. SW-MSA in particular helps to overcome the locality limitation of a single window, enabling the model to capture a wider range of contextual information and enhancing its ability to model global dependencies.

### CNN combined with ViT in medical segmentation

While both standalone convolutional neural networks (CNNs) and transformers have made progress in medical image segmentation tasks, approaches that combine the advantages of both are attracting increasing attention. For instance, TransUnet [15] uses a simplified strategy to integrate local and global features by first extracting local features through CNN convolution and then capturing global features with a transformer. Alternatively, TransFuse [25] employs a parallel branching architecture that combines the transformer and CNN in a parallel manner. This design allows the model to capture the global dependencies and low-level spatial details of both branches simultaneously, and to effectively fuse the multi-level features of the two branches through a novel fusion technique, the BiFusion module, which significantly improves the segmentation performance. The recent ParaTransCNN [16] also uses parallel CNN and Transformer encoders to effectively integrate the global information captured by the Transformer with the local features extracted by the CNN. It also introduces the SE channel attention mechanism [17] in the hopping section to highlight important features. These hybrid architectures demonstrate the advantages of combining CNN with the transformer to significantly improve the performance of medical image segmentation while maintaining computational efficiency.

## Methods

### Overall architecture

The model is designed using a two-branch structure, where one branch is based on a CNN [21], which is responsible for extracting the local details of the image, and the other branch employs Swin Transformer [13], which focuses on capturing the global contextual information of the image.

For the Swin Transformer branch, we convert an image with an input size of *H* × *W* × 3 into a feature map with a size of *H*/4 × *W*/4 × *C* by a 2D convolution (both kernel_size and stride are 4) and use it as the input to the Swin Transformer layer. After the first stage of processing, the feature map size is kept constant. In the second stage, in order to capture finer-grained features, we use the convolution operation with both kernel_size and stride of 2 to convert the feature map into a feature map with the size of *H*/8 × *W*/8 × 2*C*, which is then subjected to the Swin Transformer layer to further extract features while retaining the detail information extracted by the convolution. The third stage is similar to the second stage and continues to apply the PatchEmbedding operation and Swin Transformer processing to finally obtain an output feature map of *H*/16 × *W*/16 × 4*C*.

At the same time, the original medical images are fed into the CNN branch, which employs the pre-trained ResNet34 as the backbone to capture the local details of the images. ResNet34 [21] not only solves the gradient vanishing problem efficiently but also has less number of parameters and less computational effort.

**Fig 1:**
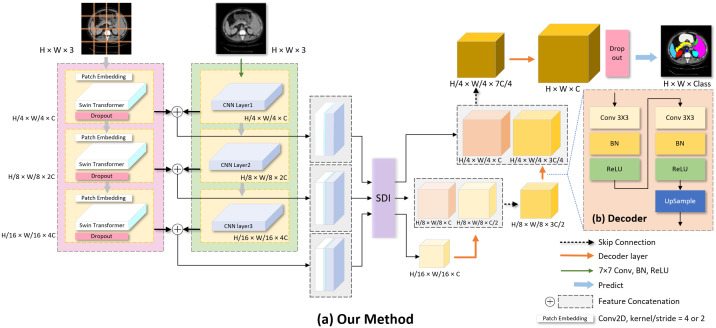
Illustration of the proposed network. **(a)** Overall architecture, including parallel CNN and Swin Transformer encoders, the SDI module in the skip connections, and the decoder. **(b)** Detailed view of the decoder architecture.

Finally, the feature maps of the two branches are fused after alignment and further refined by the SDI module [18] using multi-scale feature aggregation and attention mechanisms to obtain a richer feature representation. The fused feature maps are passed to the upsampling part of the decoder through skip connections to achieve end-to-end pixel-level prediction. We demonstrate the proposed model architecture in Fig 1.

### SwinTransformer block

**Fig 2:**
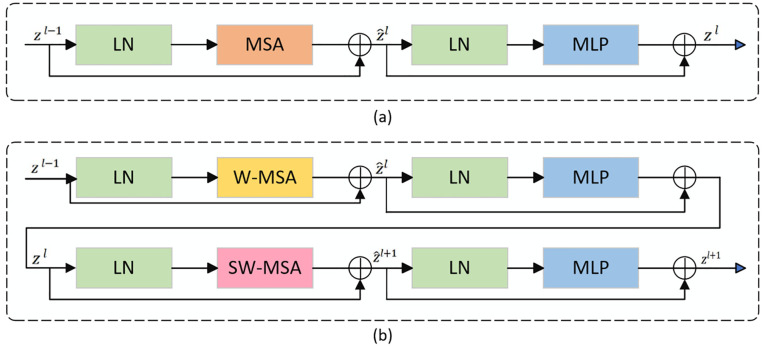
Illustration of Transformer architectures. **(a)** Standard Transformer Block. **(b)** Swin Transformer Block.

The core of the standard Transformer model consists of the Multi-head Self-Attention (MSA) module and the Multilayer Perceptron (MLP). The MSA module establishes global dependencies by calculating the attentional weights of each element in a sequence concerning all other elements, as in Fig 2a. The MLP, on the other hand, performs a nonlinear transformation of these attention-weighted features. Although the Transformer performs well in processing sequence data, its computational complexity O(*N*^2^) grows quadratically with the input sequence length, where N is the sequence length. This high complexity is particularly noticeable in intensive prediction tasks, as it requires processing many pixels or voxels, resulting in massive computational resource consumption and time cost.

To address this problem, Swin Transformer introduces two key variants: window-based multi-head self-attention (W-MSA) and shift-window-based multi-head self-attention (SW-MSA).

W-MSA divides the input feature map into fixed-size, non-overlapping localized windows and computes the multicentric self-attention independently within each window. This reduces the complexity to O($M^{2}*\frac{N}{M^{2}}=O(N)$), which is linear concerning the sequence length, where *M*^2^ is the window size and $\frac{N}{M^{2}}$ is the number of windows.

While W-MSA effectively reduces the computational burden, it lacks direct connections between windows, thus limiting its ability to model global relationships. For this reason, SW-MSA flattens windows in neighboring Swin Transformer Blocks, allowing tokens within previously different windows to be aggregated in new window divisions, thus enabling cross-window information fusion [13].

Swin Transformer gradually fuses local details with global semantic information while reducing computational complexity by alternately stacking W-MSA and SW-MSA blocks. As shown in Fig 2b, the computational flow of each Swin Transformer block is as follows:


$${\hat{z}}^{l}=\text{W-MSA}\left(\text{LN}\left(z^{l-1}\right)\right)+z^{l-1}$$ (1)

$$z^{l}=\text{MLP}\left(\text{LN}\left({\hat{z}}^{l}\right)\right)+{\hat{z}}^{l}$$ (2)

$${\hat{z}}^{l+1}=\text{SW-MSA}\left(\text{LN}\left(z^{l}\right)\right)+z^{l}$$ (3)

$$z^{l+1}=\text{MLP}\left(\text{LN}\left({\hat{z}}^{l+1}\right)\right)+{\hat{z}}^{l+1}$$ (4)

where the ${\hat{z}}^{l}$ denotes the output of the W-MSA, and *z*^*l*^ denotes the output of the MLP at layer l. Meanwhile, ${\hat{z}}^{l+1}$ denotes the output of the SW-MSA of layer l + 1, and *z*^*l*+1^ denotes the output of the MLP of layer l + 1.

Specifically, we start with an input image of size (*H* × *W* × 3). After applying a 2D convolution with a kernel size and stride size of 4, the input feature map is transformed to (*H*/4 × *W*/4 × *C*). This output then serves as an input to the Swin Transformer layer, which maintains the original spatial dimensions (*H*/4 × *W*/4) while increasing the channel count to C. In the second stage, to capture features at a finer scale, we reduce the kernel size and stride size to 2, resulting in a feature map of size (*H*/8 × *W*/8 × 2*C*). This map is then processed by the Swin Transformer layer, which extracts a more detailed representation of the features while preserving the detailed information after the convolutional transformation. The third stage mirrors the second, with continued application of Patch Embedding followed by Swin Transformer processing, culminating in an output feature map of size (*H*/16 × *W*/16 × 4*C*).

Simultaneously, the original medical images are fed into the CNN branch, which employs ResNet34 [21] as the backbone network to capture the local details of the images. We utilize a pre-trained ResNet34 model, which we will subject to ablation experiments later. This model not only addresses the gradient vanishing problem but also boasts a smaller number of parameters and lower computational requirements compared to other models.

### Semantics and detail infusion (SDI) module

#### Multi-scale feature fusion module.

Since traditional skip connections fail to capture global features at each layer, we fuse the outputs of the CNN and Swin Transformer branches after aligning their feature dimensions to leverage both global context and local details. These fused feature maps are not directly fed into the decoder but are first refined through the SDI module [18]. This module enhances semantic and detailed information by integrating multi-scale features using attention mechanisms.

**Fig 3:**
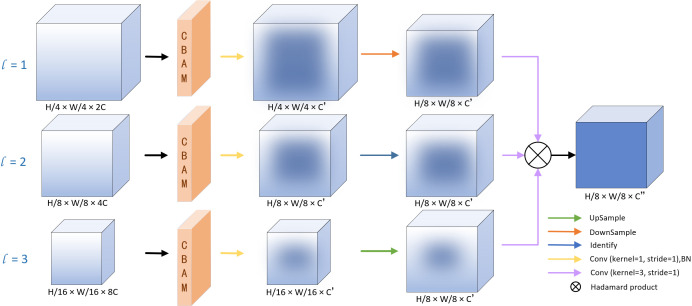
Illustration of the Semantics and Detail Infusion (SDI) module. For visualization, only the refinement of the third feature layer is shown.


As shown in Fig 3, for each SDI level j∈1,2,3, the CNN and Swin Transformer features are first concatenated along the channel dimension:

$$F_{\text{cat}}^{j}=\text{Concat}(F_{\text{CNN}}^{j},F_{\text{Trans}}^{j})\in R^{H_{j}\times W_{j}\times (C_{\text{CNN}}^{j}+C_{\text{Trans}}^{j})}.$$ (5)

The concatenated features $F_{\text{cat}}^{j}$ are first refined by CBAM [19], then passed through a 1×1 convolution layer followed by batch normalization to reduce the number of channels to c, where c is a hyperparameter set to 64 for the Synapse dataset and 96 for the AVT dataset:


$$f_{j}=BN(Conv1\times 1(CBAM(F_{\text{cat}}^{j}))).$$ (6)

To efficiently enable multi-scale fusion within the decoder, we adjust the resolution of the refined feature map *f*_*j*_ to match the resolution (*H*_*i*_, *W*_*i*_) of the target decoder stage *i*. The piecewise resizing operation is defined as follows:


$$f_{ij}=\left\{ \begin{array}{cc} D(f_{j},(H_{i},W_{i})) & \text{if }j<i,\\ I(f_{j}) & \text{if }j=i,\\ U(f_{j},(H_{i},W_{i})) & \text{if }j>i, \end{array}\right.$$ (7)

Depending on the relative positions of encoder stage j and target stage i, the resolution of the feature map is adjusted as follows:

- Downsampling (*j* < *i*): apply adaptive average pooling *D* to reduce the feature-map resolution.
- Identity mapping (*j* = *i*): apply identity mapping *I* to preserve the feature-map resolution.
- Upsampling (*j* > *i*): apply bilinear interpolation *U* to increase the feature-map resolution.

Here, *D*, *I* and *U* denote adaptive average pooling, identity mapping and bilinear interpolation, respectively.

Finally, the multi-level fusion at decoder stage *i* is computed by applying a smoothing convolution to each resized feature map and aggregating them via the Hadamard product:


$$F_{\text{fusion}}^{i}=\overset{N}{\underset{j=1}{⨀}}{\text{Conv}}_{3\times 3}(f_{ij}),$$ (8)

where ${\text{Conv}}_{3\times 3}(\cdot )$ denotes the 3 × 3 convolution operation used to smooth spatial details and refine semantic consistency, and *N* represents the number of SDI scales. This operation effectively fuses multi-scale information to enhance the decoder representation.

#### Convolutional block attention module (CBAM).

**Fig 4:**
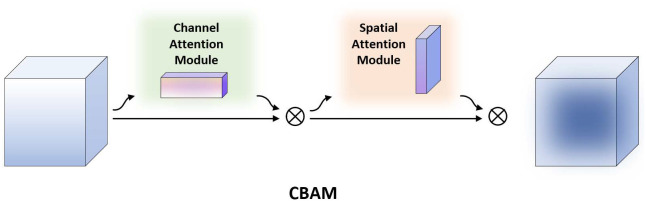
Illustration of the Convolutional Block Attention Module (CBAM).

Unlike previous SE blocks [17], which rely solely on average pooling information to excite feature channels. As shown in Fig 4, CBAM [19] sequentially applies channel and spatial attention to the input feature map and adaptively optimizes it. The entire process can be summarized as follows:


$$F^{\prime }=M_{c}(F)⊗F,\;F^{\prime \prime }=M_{s}\left(F^{\prime }\right)⊗F^{\prime }$$ (9)

where $F\in R^{C\times H\times W}$ denotes the input feature map, *M*_*c*_ and *M*_*s*_ represent the channel and spatial attention maps respectively, and ⊗ denotes element-wise multiplication.

**Fig 5:**
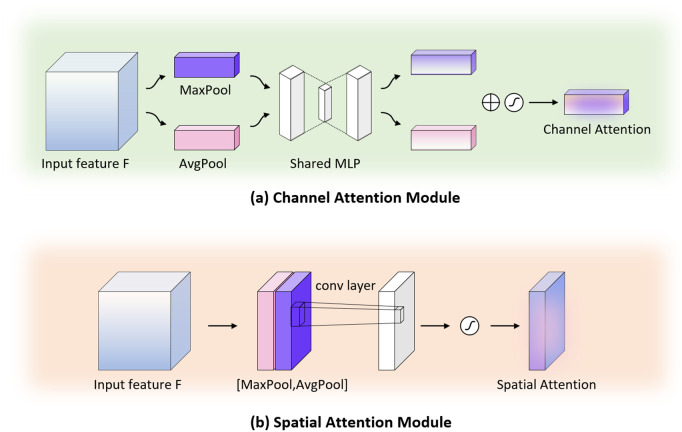
Illustration of the attention modules. **(a)** Channel Attention (CA) module. **(b)** Spatial Attention (SA) module.


As shown in Fig 5a, the channel attention module focuses on “what” is important in an image by emphasizing informative channels. To compute the channel attention map, CBAM first aggregates spatial information using both global average pooling and global max pooling [26] to produce two descriptors, $F_{c}^{avg}$ and $F_{c}^{max}$. These descriptors are then forwarded through a shared multi-layer perceptron (MLP) with one hidden layer. The channel attention map is given by:

$$M_{c}(F)=\sigma (\mathrm{MLP}(\mathrm{AvgPool}(F))+\mathrm{MLP}(\mathrm{MaxPool}(F)))$$ (10)

As shown in Fig 5b, the spatial attention module focuses on “where” is an informative part by highlighting key spatial regions. For this purpose, CBAM first applies average pooling and max pooling along the channel axis, generating two 2D maps, $F_{s}^{avg}\in R^{1\times H\times W}$ and $F_{s}^{avg}\in R^{1\times H\times W}$. These are then concatenated and passed through a convolutional layer with a kernel size of 7 × 7 to produce the spatial attention map:


$$M_{s}(F)=\sigma \left(f^{7\times 7}\left(\left[F_{s}^{avg};F_{s}^{\max}\right]\right)\right)$$ (11)

where *f*^7×7^ denotes a convolution operation with a 7 × 7 filter and the concatenation $\left[F_{s}^{avg};F_{s}^{\max}\right]$ is performed along the channel axis.

By sequentially applying these two attention modules, CBAM refines the intermediate features, allowing the network to emphasize both the semantic (channel-wise) and spatially salient regions. This attention-based feature refinement plays a crucial role in our multi-scale fusion strategy, ultimately leading to more accurate segmentation results.

### Decoder

We use skip connections in the decoder stage to connect the feature maps from the corresponding encoder stage. These connections integrate multi-scale features processed by the SDI module with the feature maps generated by the decoder. This integration enhances the semantic information and details of the feature maps in the decoder, facilitating feature propagation. We feed the integrated feature maps into each layer of the decoder, performing convolution and upsampling operations in turn. The convolutional layer is responsible for extracting the integrated multi-scale features, including 3 × 3 convolution, batch normalization, and ReLU layers, as shown in Fig 1b. The upsampling operation, on the other hand, is performed using a transposed convolutional layer to reduce the channel dimension and restore the image resolution. Specifically, we apply dropout before the feature map output, randomly disabling 50% of the nodes. This encourages independent evolution of nodes rather than co-evolution, which tends to be sub-optimal. We also performed ablation experiments to assess the impact of filtering on model performance [27,28].

## Results

In this section, we first detail the origin of the dataset and the specifics of the experimental setup, and then evaluate the performance of our proposed method on three challenging medical image segmentation tasks. We also provide an exhaustive comparison with state-of-the-art (SOTA) methods. In addition, we performed an ablation study to analyse in depth the contribution of each model component.

### Dataset

**Synapse Multi-Organ Segmentation Dataset:** We conducted our study using the dataset from the MICCAI 2015 Multi-Atlas Abdomen Annotation Challenge [29], which consists of 30 abdominal CT scans containing a total of 3779 axially enhanced clinical CT images. Each volume ranged from 85 to 198 slices, with 512 × 512 pixels per slice. The dataset provides manual annotations for eight abdominal organs, including the liver, spleen, pancreas, aorta, gallbladder, stomach, left kidney, and right kidney. Following previous studies [14,15], 18 cases were used for training and the remaining 12 cases were used for testing.

**Aortic Vessel Tree (AVT) Segmentation Dataset:** We used 56 CTA scans from the SEG.A. challenge 2023 [30]. The datasets were obtained from three medical centres: the KiTS Grand Challenge dataset (K dataset, 20 cases), the Rider Lung CT dataset (R dataset, 18 cases) and the Dongyang Hospital dataset (D dataset, 18 cases). To enable more detailed analysis, all image slices were resampled to an isotropic spatial resolution of 1 mm × 1 mm, and the Hounsfield Unit (HU) values were normalized to the range [0, 1]. Following previous study, 38 cases (15,044 slices) were randomly assigned to the training set, while the remaining 18 cases were used for testing.

### Implementation details

All experiments were conducted on a single NVIDIA RTX A100 GPU using a software environment consisting of CUDA 11.7, PyTorch 1.21.1, and Python 3.9.19. The input image size for model training was uniformly set to 224 × 224, and the batch size was set to 32. We performed 250 epochs of training using the AdamW [31] optimizer with an initial learning rate of 0.001. The default random seed was set to 1234 for all experiments. During the training process, a cosine annealing strategy was used to adjust the learning rate, which helped the model stabilisation and weight refinement. The loss function combined dice loss and cross-entropy loss, $\lambda_{1}$ and $\lambda_{2}$ represent different weight coefficients, which are set to 0.5 and 0.5 respectively in the experiments, and their expressions are as follows:


$$ℒ=\lambda_{1}{ℒ}_{Dice}+\lambda_{2}{ℒ}_{CE}$$ (12)

In order to improve the transfer learning ability [32] and initialisation performance [33] of the model in medical image segmentation tasks, we initialized the parameters using pre-training weights from ImageNet for both CNN and Swin Transformer modules. To enhance the model’s adaptability to image variations, we also introduced data enhancement techniques during training, including random image flipping and rotation. To ensure a fair “apples-to-apples” comparison, we adopted the official dataset splits and identical pre-processing pipelines for all methods. While the baseline ParaTransCNN [16] was retrained using its officially recommended optimal hyperparameters to reach its peak performance, other baseline results were directly cited from its literature as they were produced under the same data protocols. We believe that using model-specific optimal hyperparameter configurations (e.g., batch size etc.) provides a more rigorous evaluation of each architecture’s true potential. To prevent overfitting, we applied dropout [28] within the Swin Transformer to reduce feature noise and unnecessary details, and also before the final output to ensure model output stability. In addition, we conducted a series of ablation experiments to verify the impact of each component on the model performance.

### Assessment of results

We used task-specific assessment metrics in each experiment. Specifically, these metrics included Dice coefficient(also known as the Sørensen-Dice coefficient, F1-score, DSC) and Hausdorff Distance (HD), and for boundary analysis, Average Surface Distance (ASD) and Average Symmetric Surface Distance (ASSD).

**Dice coefficient (DSC)** is a metric that measures the similarity between two samples, typically used to assess the similarity between the ground truth and predicted masks (values range from 0 to 1). The formula is as follows:


$$\text{Dice}=\frac{2|X\cap Y|}{\left|X|+|Y\right|}$$ (13)

where |*X*| and |*Y*| denote the ground truth and the predicted mask of the segmentation. The closer the Dice coefficient is to 1, the higher the overlap between the model predictions and the actual labels, indicating better model performance.

**Hausdorff Distance (HD)** measures the distance between two ensembles, specifically the 95th percentile of distances between boundary points in the ground truth and prediction ensembles. It aims to minimize the impact of outliers. The smaller the HD, the better the segmentation performance.

For a more rigorous evaluation of the quality of the predicted boundaries, we additionally report the following surface-based metrics:

**Average Surface Distance (ASD)** calculates the average of the distances from every point on the predicted surface *S*_*Y*_ to the closest point on the ground truth surface *S*_*X*_. A smaller value indicates a better fit of the predicted boundary to the ground truth.

**Average Symmetric Surface Distance (ASSD)** is the average of the two directional average surface distances, ensuring the error is measured symmetrically from both the predicted surface and the ground truth surface. This metric provides a robust measure of the average boundary error. A smaller ASSD (closer to 0 mm) indicates superior segmentation accuracy at the object boundary.

#### Result on synapse multi-organ segmentation.

**Fig 6:**
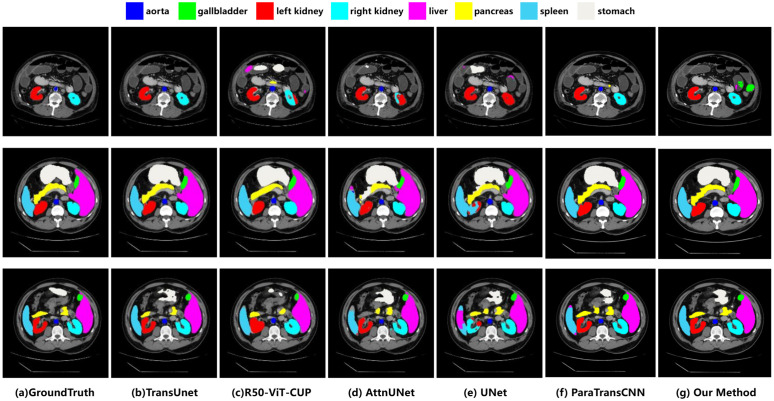
Visual comparison of Synapse Multi-organ segmentation (from TransUnet [15]).

We compared our proposed method with the previous state-of-the-art (SOTA) method in terms of average DSC and average HD for the eight abdominal organs. As shown in Table 1, our method outperformed other single-modal methods in both metrics, achieving the highest mean DSC of 84.64% and the lowest mean HD of 12.95 mm among many excellent models. In particular, it showed significant improvement in DSC for the right kidney and pancreas segmentation, improving on the original best results by 1.27% and 1.81%, respectively. Furthermore, we visualize the segmentation performance of our method for each organ using 2D representations in Fig 6.

**Table 1:** The comparison of segmentation results on Synapse Multi-Organ Segmentation between the proposed method and other models.

Methods	DSC(%)	HD(mm)	DSC(%)							
			AO	GB	LK	RK	Liv	Pa	Sp	Sto
R50 U-Net [15]	74.68	36.87	87.47	66.36	80.60	78.19	93.74	56.90	85.87	74.16
UNet [6]	76.85	39.70	89.07	69.72	77.77	68.60	93.43	53.98	86.67	75.58
R50 Att-UNet [15]	75.57	36.97	55.92	63.91	79.20	72.71	93.56	49.37	87.19	74.95
Att-UNet [22]	77.77	36.02	**89.55**	68.88	77.98	71.11	93.57	58.04	87.30	75.75
R50 ViT[15]	71.29	32.87	73.73	55.13	75.80	72.20	91.51	45.99	81.99	73.95
TransUNet [15]	77.48	31.69	87.23	63.13	81.87	77.02	94.08	55.86	85.08	75.62
TransNorm [34]	78.40	30.25	86.23	65.10	82.18	78.63	94.22	55.34	89.50	76.01
Swin UNet [14]	79.13	21.55	85.47	66.53	83.28	79.61	94.29	56.58	90.66	76.60
TransDeeplab [35]	80.16	21.25	86.04	69.16	84.08	79.88	93.53	61.19	89.00	78.40
HiFormer [36]	80.39	14.70	86.21	65.69	85.23	79.77	94.61	59.52	90.99	81.08
VM-UNet [37]	81.08	19.21	86.40	69.41	86.16	82.76	94.17	58.80	89.51	81.40
MISSFormer [38]	81.96	18.20	86.99	68.65	85.21	82.00	94.41	65.67	91.92	80.81
TransCeption [39]	82.24	20.89	87.60	71.82	86.23	80.29	95.01	65.27	91.68	80.02
DAE-Former [40]	82.43	17.46	88.96	72.30	86.08	80.88	94.98	65.12	91.94	79.19
ParaTransCNN [16]	83.49	16.06	88.61	72.26	87.97	82.83	94.82	68.59	90.55	**82.31**
**Our Method**	**84.19 ± 0.49**	**12.64 ± 1.86**	89.37 ± 0.39	**73.15 ± 2.76**	**88.40 ± 0.43**	**85.26 ± 0.46**	**95.29 ± 0.11**	**68.99 ± 2.48**	**91.16 ± 1.39**	81.86 ± 0.82

* Note: Bold values represent the best performers. Abbreviation: AO, Aorta; GB, Gallbladder; LK, Left Kidney; RK, Right Kidney; Liv, Liver; Pa, Pancreas; Sp, Spleen; Sto, Stomach.

It can be intuitively seen that UNet [6] has limitations in segmentation and is unable to accurately determine the position of the left and right kidneys, leading to incorrect segmentation of the left and right kidneys as well as the liver. Attention U-Net [22] adds an attention mechanism to UNet, but in pancreas segmentation still produces coarse boundary predictions, leading to incomplete and incorrect segmentation results. TransUnet [15] and R50-ViT-CUP [15] merge the strengths of the CNN and Transformer architectures to improve segmentation performance, but Transformer may not be as robust as CNN in exploiting local features, resulting in limited performance in tasks that require fine-grained local features. As can be seen in the third row of the figure, both predictions of pancreatic organs are coarse in terms of boundaries and shapes. More seriously, R50-ViT-CUP incorrectly fills the left renal internal foramen.

In addition, we conducted a detailed comparison with ParaTransCNN [16]. While ParaTransCNN achieves competitive overall scores, localized mis-segmentations are still observed in complex regions. As shown in the third slice of Fig 6, ParaTransCNN inaccurately predicts the spleen boundary, misidentifying splenic tissue as the liver.

**Fig 7:**
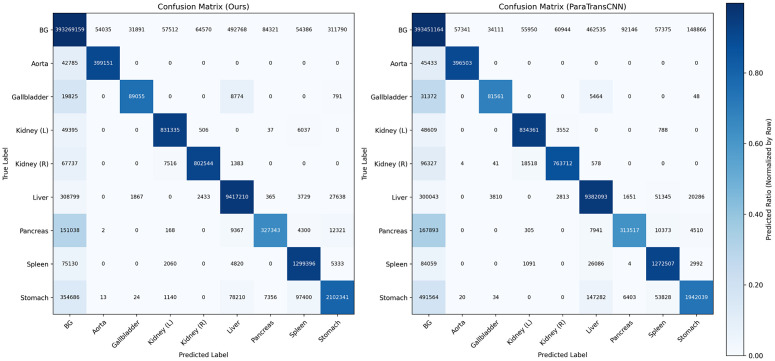
Per-class confusion matrices of the proposed method (a) and ParaTransCNN [16] (b) on the Synapse dataset. Brighter diagonal elements indicate higher class-specific segmentation accuracy.

This qualitative observation is further supported by the pixel-level confusion matrix analysis (see Fig 7). The confusion matrix reveals that ParaTransCNN misclassifies 26,086 pixels of the spleen as liver, whereas our method significantly reduces this specific error to only 4,820 pixels. Furthermore, in the pancreas segmentation, our model achieves a DSC of 68.99 ± 2.48%, outperforming ParaTransCNN’s 68.59%. The confusion matrix confirms this improvement, showing that our model correctly identifies 327,343 pancreatic pixels compared to ParaTransCNN’s 313,517. These results, along with the variance measures (Mean ± SD) and the significantly lower Hausdorff Distance (12.64 mm vs. 16.06 mm) in Table 1, demonstrate our model’s superior precision and robustness in handling fine anatomical structures and organ boundaries.

#### Result on AVT segmentation.

The results of our proposed method’s performance on the AVT dataset, as shown in Table 2, demonstrate that it outperforms its competitors in most evaluation metrics. Specifically, our model’s dominance on different datasets highlights its satisfactory generalization ability.

**Table 2:** The comparison of segmentation results on AVT medical images between the proposed method and other models.

Methods	All dataset		K dataset		R dataset		D dataset	
	DSC(%)	HD(mm)	DSC(%)	HD(mm)	DSC (%)	HD(mm)	DSC(%)	HD(mm)
UNet [6]	82.89	7.27	79.22	8.83	76.32	10.34	93.13	2.65
Att-UNet [22]	80.25	9.45	77.91	9.58	70.15	15.18	92.69	3.58
TransUNet [15]	82.99	11.43	80.67	9.61	74.73	23.47	93.59	1.21
Swin UNet [14]	79.62	13.76	77.45	12.62	69.41	26.48	92.02	2.19
TransDeeplab [35]	79.72	12.95	75.15	12.06	71.06	24.88	92.97	1.90
HiFormer [36]	82.53	7.35	78.69	9.45	75.56	11.19	93.35	1.41
MISSFormer [38]	79.09	12.25	75.81	12.45	69.20	21.62	92.28	2.67
TransCeption [39]	82.87	10.33	79.37	11.97	75.54	17.92	93.70	1.21
DAE-Former [40]	85.02	8.96	82.25	11.37	78.85	14.32	93.96	1.21
ParaTransCNN [16]	87.27	**5.31**	85.76	7.32	82.06	**7.55**	**93.99**	**1.07**
**Our Method**	**87.91 ± 0.21**	7.06 ± 1.25	**85.90 ± 0.31**	**6.28 ± 1.09**	**83.97 ± 0.74**	13.63 ± 3.14	93.88 ± 0.16	1.08 ± 0.06

* Note: Bold values represent the best performers.

**Fig 8:**
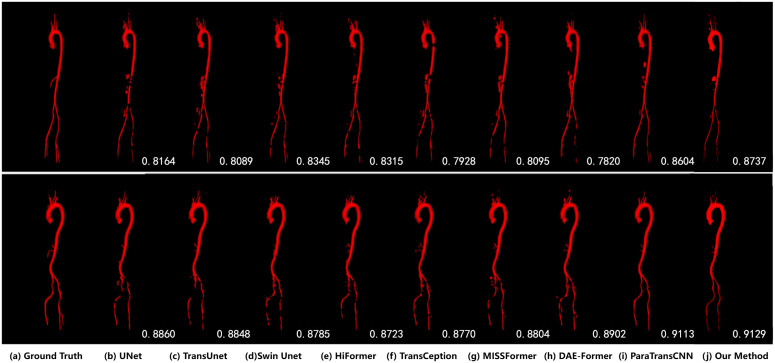
Visual comparison of Aortic Vessel Tree segmentation (from ParaTransCNN [16]).

We also provide a visual comparison of AVT segmentation in Fig 8. It is evident that our approach, which utilizes parallel CNN and Transformer, can discern more detailed features, enabling us to capture finer structures and generate more accurate contours. The expedient combination of CNN and Transformer to model global relationships and local representations results in superior performance.

#### Boundary quality and surface distance metrics.

To provide a rigorous assessment of our model’s localization precision, particularly at object boundaries, we report the surface-based metrics, Average Surface Distance (ASD) and Average Symmetric Surface Distance (ASSD), in Table 3. These metrics reflect the average deviation between the predicted and ground truth surfaces, where lower values indicate tighter boundary adherence.

**Table 3:** Comparison of surface-based boundary metrics on the Synapse and AVT datasets.

Methods	Synapse dataset		AVT dataset	
	ASD(mm)	ASSD(mm)	ASD(mm)	ASSD(mm)
TransUNet [15]	8.88	5.30	2.75	2.02
ParaTransCNN [16]	5.17	3.14	**1.62**	**1.34**
**Our Method**	**3.85**	**2.40**	1.80	1.35

* Note: Bold values represent the best performers.

The results show that our method achieves the best boundary fidelity in the multi-organ segmentation task (Synapse dataset), yielding the lowest ASD (3.85 mm) and ASSD (2.40 mm), which significantly surpasses all competitive methods. On the Aortic Vessel Tree (AVT) dataset, which demands high structural continuity, our method achieves an ASSD (1.35 mm) that is highly competitive with the ParaTransCNN (1.34 mm).

#### Comparison of model parameters.

Among the many well-performing models, we selected the top ones for parameter comparison with our proposed method. As shown in Table 4, our method maintains excellent performance while significantly reducing the number of parameters. Compared to TransUNet (105.28M), Swin-Unet (149.22M), and ParaTransCNN (234.10M), our method uses only 22.52M parameters, reducing them by approximately 78.6%, 84.9%, and 90.4%, respectively. This is especially important for application scenarios where models need to be deployed on resource-constrained devices.

**Table 4:** Comparison of the number of parameters, FLOPs, and performance across models in the Synapse Multi-Organ Segmentation.

Method	Params(M)	FLOPs(G)	DSC(%)	HD(mm)
TransUnet [15]	105.28	24.66	77.48	31.69
Swin-Unet [14]	149.22	30.14	79.13	21.55
ParaTransCNN [16]	234.10	529.09	83.49	16.06
Att-UNet [22]	34.90	101.90	77.77	36.02
MISSFormer [38]	42.46	54.46	81.96	18.20
**Our Method**	**22.52**	**20.57**	**84.19 ± 0.49**	**12.64 ± 1.86**

#### Computational efficiency analysis.

To evaluate the clinical feasibility of our model, we recorded the computational costs on the Synapse and AVT datasets. As presented in Table 5, our method achieves notable improvements in both training efficiency and inference throughput compared with the competing methods ParaTransCNN and TransUnet. Notably, thanks to the lightweight nature of our architecture, we were able to utilize significantly larger batch sizes (e.g., 32 and 20 for Synapse and AVT, respectively) compared to the ParaTransCNN method (Batch Size = 4), thereby drastically accelerating the training process.

**Table 5:** Computational efficiency and resource consumption on different datasets.

Dataset	Method	GPU Memo(G)	Training Time (min/epoch)	Total Wall-clock Time(h)	Inference Speed (slices/sec)
**Synapse**	TransUnet [15]	8.60	0.83	2.07	0.83
	ParaTransCNN [16]	14.19	2.96	7.39	1.94
	**Ours**	**6.58**	**0.28**	**1.18**	**1.98**
**AVT**	TransUnet [15]	8.60	4.62	11.55	8.89
	ParaTransCNN [16]	14.18	16.28	40.71	14.24
	**Ours**	**5.92**	**2.65**	**7.52**	**17.39**

* Note: To maximize throughput and demonstrate model efficiency, the Batch Size (BS) for our method was set to 32 and 20 for Synapse and AVT datasets, respectively. The baseline ParaTransCNN and TransUnet were limited to BS = 4 and BS = 24 due to their higher memory footprint.

On the Synapse dataset, the training time per epoch decreases from 2.96 minutes (ParaTransCNN) to 0.28 minutes, reflecting a substantial reduction in computational overhead. Likewise, on the large-scale AVT dataset, the total training duration is shortened from more than 40 hours to 7.5 hours, demonstrating the scalability of our framework. In terms of inference, our model consistently delivers higher throughput; on the AVT dataset, it attains an inference rate of 17.39 slices/sec, surpassing both comparison methods and enabling rapid response in clinical workflows. Furthermore, our method reduces GPU memory consumption by approximately 53% and 58% on the Synapse and AVT datasets, respectively, enhancing its suitability for deployment on resource-limited hardware platforms.

### Ablation study

In this section, we will conduct experiments on the Synapse dataset to further ablation studies for this segmentation task to evaluate the capability of each component in the proposed model. To ensure a fair comparison and eliminate the impact of random initialization, all experiments in this section were conducted with the random seed fixed to 1234.

#### Comparing different layers of CNN backbone networks.

We first examined the contribution of different layers of the CNN backbone. Specifically, we used a series of ResNet variants as a convolutional architecture. As shown in the second row of Table 6, the best performance is obtained by utilizing the ResNet34 backbone. Moreover, we can see that a larger CNN backbone does not necessarily lead to performance improvements (see the fourth row in the table), which led us to use ResNet34 architecture as the default CNN architecture.

**Table 6:** Comparing Different Layers of CNN Backbone Networks. Except for the CNN module, all configurations are identical to our proposed method.

Methods	Params(M)	FLOPs (G)	DSC(%)	HD(mm)
ResNet18	**12.88**	**13.66**	83.19	15.46
**ResNet34**	22.52	20.57	**84.64**	**12.96**
ResNet50	25.54	22.39	84.27	15.83
ResNet101	43.66	36.29	83.14	19.19

#### Impact of the skip module.

Next, we evaluate the importance of the SDI module at the skip connection for segmentation performance. In addition, we conduct a detailed assessment of individual components within the SDI module. As seen in Table 7, the SDI module significantly improves performance metrics, including DSC and HD.

**Table 7:** Results of ablation experiments for each component of the network model.

Methods	Params(M)	FLOPs (G)	DSC(%)	HD(mm)
Baseline	50.22	72.91	83.51	16.26
+MSFF	**22.43**	20.57	83.91	14.36
**+MSFF+CBAM**	22.52	**20.57**	**84.64**	**12.96**

* Note: MSFF denotes the multi-scale feature fusion module, which is the structure remaining after the spatial detail module excludes CBAM.

We use the encoder of parallel Swin Transformer and ResNet34 as a baseline network (Baseline) and gradually add the proposed components at skip connections to demonstrate their effectiveness. The results of these ablation experiments are presented in Table 7.

A comparative analysis of the experimental outcomes reveals substantial advancements in model performance through strategic architectural modifications. First, the implementation of the multi-scale feature fusion(MSFF) strategy demonstrates dual benefits in model efficiency and segmentation accuracy. Specifically, this approach achieves a 55.3% reduction in parameter count (50.22M to 22.43M) while simultaneously elevating the Dice similarity coefficient (DSC) by 0.40% (83.51% to 83.91%) and reducing Hausdorff distance (HD) by 1.90 mm (16.26 mm to 14.36 mm). This dimensional modulation-driven optimization, whose efficacy is systematically validated through grid search experiments in Section, establishes an effective balance between computational complexity and representational capacity. Subsequent integration of the CBAM attention mechanism yields further performance gains, boosting DSC by 0.73% (83.91% to 84.64%) and substantially decreasing HD by 9.7% (14.36 mm to 12.96 mm). Notably, the final optimized architecture achieves cumulative enhancements of 1.13% in DSC and 3.30 mm reduction in HD relative to baseline, accompanied by a remarkable 55.1% parameter reduction (50.22M to 22.52M). These empirical results confirm that the integrated SDI module realizes synergistic optimization through multi-component integration, enabling simultaneous breakthroughs in segmentation accuracy and operational robustness while maintaining computational efficiency, as evidenced by stabilized FLOPs at 20.57G.

#### Impact of the number of head.

In Swin Transformer, the number of heads is an important parameter in the Multi-Head Self-Attention (MHSA) mechanism. The Multi-Head Attention mechanism allows the model to distribute attention across various representation subspaces simultaneously, enhancing its capacity to capture diverse information. Each head within the mechanism performs self-attention independently, and the outputs are then combined and integrated through a subsequent linear layer.

From Table 8, it can be seen that increasing the number of heads by a moderate amount can improve the performance of the model. For example, when the number of heads is increased from [4,4,4] to [8,8,8], the DSC improves from 82% to 84.64%, and the HD decreases from 15.82 mm to 12.96 mm. However, comparing the data in the second and fourth rows, increasing the number of heads from [8,8,8] to [16,16,16] shows that an excessive number of heads does not necessarily improve performance and may increase computational complexity. Specifically, the configuration [16,16,16] leads to a higher GPU memory usage (6.77G) and marginally higher FLOPs (+0.03%), yet results in a performance drop. The configuration [8,8,8] is therefore selected as the final setting, representing the optimal trade-off between segmentation accuracy and practical computational cost (GPU memory utilization).

**Table 8:** Results of ablation experiments for the number of heads. For a fair comparison, ResNet34 has been used as the CNN module in all the configurations.

Method	Dim	Head	Params(M)	FLOPs(G)	ΔFLOPs(%)	GPU(G)	DSC(%)	HD(mm)
**Ours**	64	[4,4,4]	**22.52**	**20.57**	**0.00%**	**6.49**	82.00	15.82
	64	[4,8,16]	22.52	20.57	+0.00%	6.52	82.90	14.55
	**64**	**[8,8,8]**	22.52	20.57	+0.01%	6.58	**84.64**	**12.96**
	64	[16,16,16]	22.52	20.57	+0.03%	6.77	83.74	14.93

* Note: FLOPs reported with full precision: [20.56775, 20.56999, 20.56849, 20.57446] GFLOPs for [4,4,4], [8,8,8], [4,8,16], [16,16,16], respectively. ΔFLOPs (%) is relative to [4,4,4]. Although theoretical FLOPs differences are small, GPU memory usage provides additional practical cost metrics.

#### Impact of the token dimensions.

Token dimensions in the transformer architecture are critical for capturing global information, which determines the amount of information the model can store for each token. A higher token dimension provides a richer representation of features, which helps the model to capture more complex patterns and relationships. In some cases, increasing token dimension can improve the performance of the model, especially when dealing with tasks that require rich feature representations. However, this performance improvement may plateau after a certain point, as the gains from increasing dimensionality are counterbalanced by higher computational cost.

Therefore, we varied the number of token dimensions while keeping hyperparameters like batch size fixed, to explore their impact on model performance. We tested token dimensions of 32, 64, 96, and 128. As shown in Table 9, the model achieves its highest performance when the token dimension is set to 64 (DSC of 84.64%). While increasing the dimension to 96 or 128 (resulting in DSC of 82.70% and 83.74%, respectively) does not yield consistent performance improvements, it comes with a significantly higher computational burden. Specifically, the Dim = 128 configuration increases FLOPs by over 176% and GPU memory usage by nearly 85% compared to Dim = 32. The selection of Dim = 64 thus represents the optimal trade-off between segmentation accuracy and computational cost, allowing us to maintain high performance without excessive resource consumption.

**Table 9:** Results of ablation experiments on token dimension.

Method	Dim	Head	Params(M)	FLOPs(G)	ΔFLOPs(%)	GPU(G)	DSC(%)	HD(mm)
**Ours**	32	[8,8,8]	**20.91**	**15.19**	**0.00%**	**5.05**	82.59	22.60
	**64**	**[8,8,8]**	22.52	20.57	+35.46%	6.58	**84.64**	**12.96**
	96	[8,8,8]	25.17	29.52	+94.42%	7.94	82.70	21.36
	128	[8,8,8]	28.85	42.04	+176.87%	9.33	83.74	17.32

#### Impact of the dropout.

Dropout is used as a regularization technique to reduce overfitting by randomly discarding neurons during training [28]. To investigate the regularization effects of Dropout in different network stages, we conduct systematic ablation studies with a 50% dropout rate applied at two critical positions: (a) after Swin Transformer feature extraction and (b) before final prediction output. The experimental configurations and corresponding performance metrics are detailed in Table 10.

**Table 10:** Results of ablation experiments for dropout.

Method	Position		DSC(%)	HD(mm)
	SwinTransformer	Output		
**Dropout**	0	0	83.08	16.84
	0	0.5	83.08	17.17
	0.5	0	84.02	**11.37**
	**0.5**	**0.5**	**84.64**	12.96

The experimental results show that applying Dropout only before the predicted output maintains the DSC (83.08%) but leads to a slight increase in the boundary error (HD: 17.17 mm), suggesting that the end-randomized mask may interfere with the spatial continuity of the anatomical structure. On the other hand, applying 50% Dropout after Swin Transformer feature extraction significantly improves the model performance, increasing the Dice coefficient from the baseline 83.08% to 84.02% and reducing the HD by 32.5% (16.84 mm → 11.37 mm), confirming that this strategy can effectively inhibit the overfitting of global contextual features and enhance the boundary localization accuracy. In addition, the highest DSC (84.64%) is obtained if the two-stage Dropout is combined. Still, its HD (12.96 mm) is elevated by 13.9% compared with the single-feature-layer Dropout, which suggests that the model improves the robustness of the region identification while slightly compromising the boundary consistency of the fine-grained anatomical structure.

In summary, applying Dropout after feature extraction can best suppress overfitting and improve segmentation quality; if we further pursue higher DSC, we can additionally add Dropout at the output on this basis, but we should weigh its slight impact on boundary consistency.

### More experiments

#### Different selection on optimizer.

Optimizers play a crucial role in machine learning and deep learning. Their main role is to tune the parameters of the model to minimize the loss function and thus improve the performance of the model. Among these, Stochastic Gradient Descent (SGD) [41], a widely used optimization algorithm, updates model weights by randomly selecting samples and can sometimes find a better global minimum. However, it has several hyperparameters, such as learning rate, momentum, etc., and choosing the appropriate hyperparameters is crucial. Considering these factors, we chose the AdamW[31] optimizer for this study. AdamW builds on the original Adam [42] optimizer by introducing weight decay, addressing regularization issues, and allowing for faster convergence with less sensitivity to hyperparameter selection.

As seen in Table 11, when combined with the AdamW optimizer and a cosine annealing learning rate decay strategy, it achieves excellent performance on the Synapse image segmentation task. This suggests that by carefully choosing the optimizer and adjusting the training strategy, the model’s learning and generalization capabilities can be significantly improved to achieve more accurate image segmentation results.

**Table 11:** Results of ablation experiments for learning rate tuning strategy and optimizer selection.

Optimizer	LR Schedule	DSC(%)	HD(mm)
SGD	poly	76.08	38.93
SGD	cosine	76.80	40.65
**AdamW**	**cosine**	**84.64**	**12.96**

#### Different image input size.

In practice, in order to strike a balance between the computational complexity of the model and prediction accuracy, we usually adopt a step-by-step strategy. Initially, we use lower resolution images for the initial segmentation prediction. The advantage of this approach is that it can generate results quickly while significantly reducing computational resource requirements. However, the use of lower resolution images may result in the loss of some detailed information, thus affecting segmentation accuracy.

**Fig 9:**
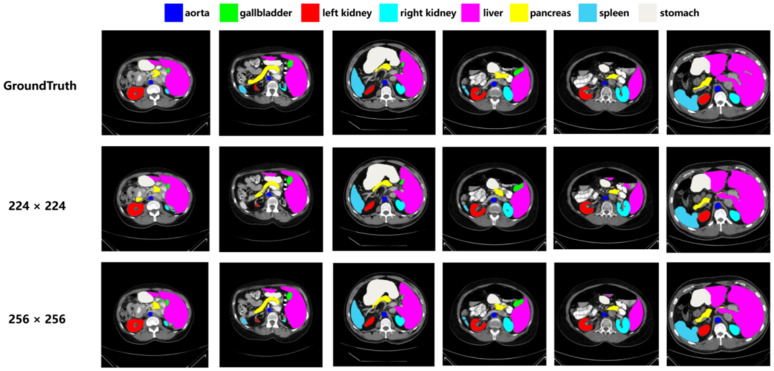
Segmentation effect of the model on different input image resolutions.

Specifically for the data in Table 12, when the input image size is increased from 224 pixels to 256 pixels, the DSC of the model increases from 84.64% to 85.97%, while the HD decreases from 12.96 mm to 9.6 mm. More intuitively, Fig 9 shows that an increased input resolution captures finer anatomical structures, leading to more accurate predicted segmentation results.

**Table 12:** Segmentation performance of the model on different input image resolutions.

Image Size	DSC(%)	HD(mm)	DSC(%)							
			AO	GB	LK	RK	Liv	Pa	Sp	Sto
224×224	84.64	12.96	89.25	73.16	88.22	85.11	95.26	71.60	91.78	82.73
**256×256**	**85.97**	**9.60**	**90.08**	**74.41**	**89.59**	**86.11**	**95.65**	**74.72**	**92.83**	**84.39**

## Discussion

Comprehensive experimental results across multiple medical image segmentation datasets demonstrate that our model outperforms CNN and Transformer-based methods, surpassing existing state-of-the-art (SOTA) methods in most cases. This superior performance validates the effectiveness of our proposed approach. The advantages of our model are twofold. First, its parallel encoding structure, combining CNN and Swin Transformer, enables it to capture local and global key information. Second, the SDI module integrates multi-scale features through attention mechanisms, enhancing useful information while suppressing irrelevant details, leading to accurate segmentation and precise localization. Quantitatively, our model achieves the best overall performance on the multi-organ dataset and excels in segmenting multiple organs. Notably, it improves Dice coefficients by 7.34% and 5.02% over UNet on multi-organ and aortic vessel tree segmentation tasks, respectively. Furthermore, the model’s lightweight design (22.52M) significantly reduces computational overhead compared to ParaTransCNN (234.10M) and Swin-Unet (149.22M), making it feasible for deployment in resource-constrained clinical environments.

### Clinical significance

The proposed method holds substantial promise for clinical applications. Accurate segmentation of anatomical structures (e.g., aortic vessel trees) can directly assist radiologists in diagnostic workflows by reducing manual annotation time and improving reproducibility. For instance, the achieved Dice coefficient of 87.91% on aortic vessel tree segmentation could enhance preoperative planning for endovascular interventions by providing precise vascular maps. Additionally, the model’s efficiency enables integration into real-time systems, such as intraoperative navigation tools, where rapid segmentation updates are critical. The lightweight design further supports deployment on portable devices in resource-limited settings, broadening access to advanced diagnostic tools. Future integration with explainability frameworks could also foster clinician trust by visualizing attention maps highlighting decision-critical regions (e.g., tumor boundaries in low-contrast CT slices).

### Boundary quality and failure analysis

The analysis of surface distance metrics (Table 3) provides crucial insights into our model’s localization capabilities. The superior ASD and ASSD scores on the Synapse dataset validate the effectiveness of our parallel encoder in handling the low-contrast, complex boundaries typical of abdominal organs (e.g., pancreas, spleen). Our model achieves high precision in these challenging delineation tasks.

On the AVT dataset, where extreme structural continuity is critical, the minimal difference in ASSD (1.35 mm vs. 1.34 mm for ParaTransCNN) represents an acceptable trade-off. We attribute the slightly higher ASD (1.80 mm) primarily to the accumulation of average errors at the distal ends of minute vascular branches. Through examination of the specific failure cases, these errors are identified as typically small False Negatives resulting from the Partial Volume Effect (PVE).

This minimal trade-off in mean surface accuracy is justified by our model’s overall best performance in the volumetric Dice coefficient and, more importantly, by its substantial computational efficiency. The combination of high overall accuracy and lightweight design demonstrates a superior performance-to-cost ratio, enhancing its clinical deployability.

### Limitations

Despite these strengths, our approach has several limitations. First, our current evaluation is focused on 2D slice-based segmentation. We recognize the importance of 3D processing, but a direct extension of our current CNN-Transformer architecture to true 3D volumetric inputs presents significant challenges and computational costs. Naively extending the Swin Transformer to 3D (i.e., using 3D windowed attention) would lead to prohibitive GPU memory requirements, as the complexity of self-attention scales cubically with volume size (*O*(*N*^3^)). This high computational barrier directly conflicts with our goal of maintaining a lightweight framework suitable for resource-constrained clinical deployment. Therefore, our future research will focus on exploring memory-efficient 3D strategies (such as optimized 2.5D methods or dedicated 3D attention mechanisms) to find an optimal balance between volumetric segmentation accuracy and practical deployment cost. Second, the model’s performance relies on the quality and diversity of the training data. While our model shows robustness on public benchmarks, its generalization to highly heterogeneous clinical data with ambiguous or noisy annotations remains to be further validated. Integrating self-supervised learning could be a promising direction to mitigate this by leveraging unlabeled data to enhance feature representation learning.

## Supporting information

S1 Fig The segmentation failure cases of the pancreatic organ. In columns 2, 4, and 6, the red regions represent FN (False Negative), the blue regions represent FP (False Positive), and the green regions represent TP (True Positive); in columns 3, 5, and 7, the red lines represent FN, and the green lines represent TP.
(PDF)

S2 Fig The segmentation failure cases of small vessels. In columns 2, 4, and 6, the red regions represent FN (False Negative), the blue regions represent FP (False Positive), and the green regions represent TP (True Positive); in columns 3, 5, and 7, the red lines represent FN, and the green lines represent TP.
(PDF)

S1 Table Quantitative comparison of Dice score and HD95 on the Synapse dataset across different random seeds. (PDF)

S2 Table Quantitative results (Mean ± SD) and paired significance tests on the Synapse dataset. (PDF)

S3 Table Quantitative comparison of Dice score and HD95 on the AVT dataset across different random seeds. (PDF)

S4 Table Quantitative results (Mean ± SD) and paired t-tests on the AVT dataset subsets. (PDF)
