# Supplementary figures and images for: A hybrid CNN-Transformer network integrating multiscale spatially detailed features for medical image segmentation
Source: PLoS One. 2026 Apr 29;21(4):e0345549. doi: 10.1371/journal.pone.0345549 (PMC13128111; doi:10.1371/journal.pone.0345549)

(a) GroundTruth

(b) Ours

(c) ParaTransCNN

(d) TransUnet

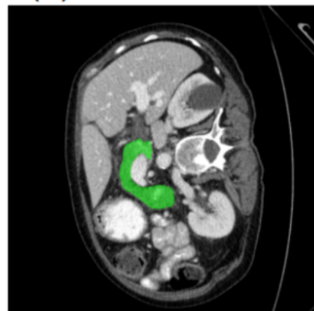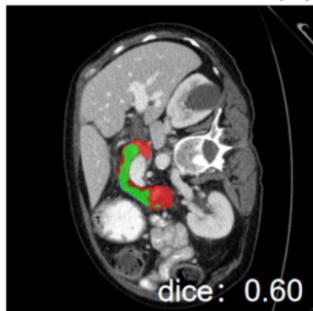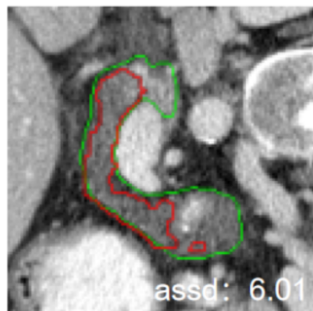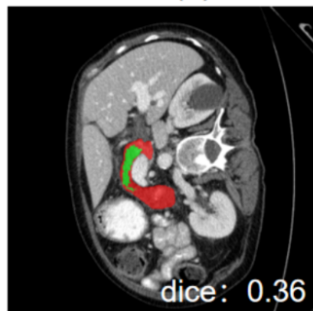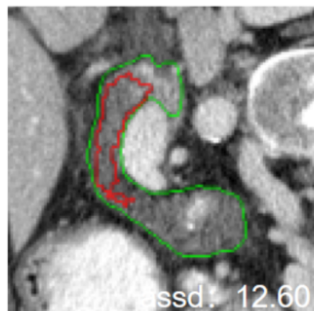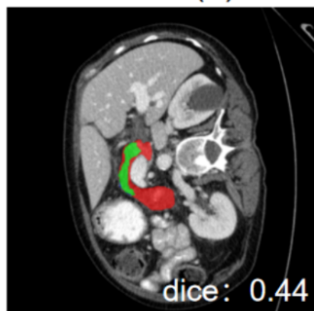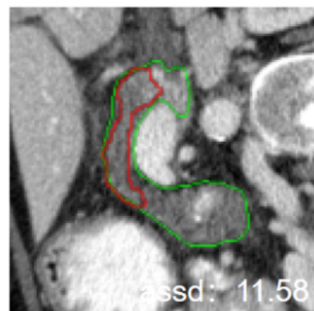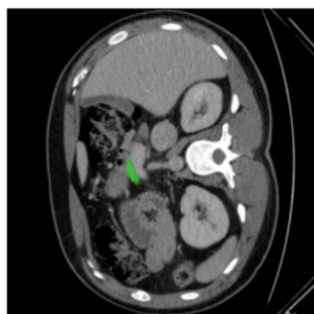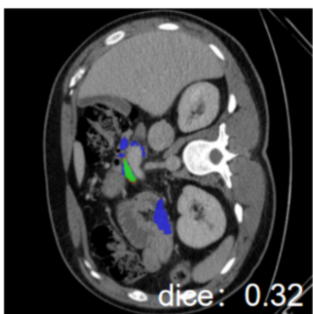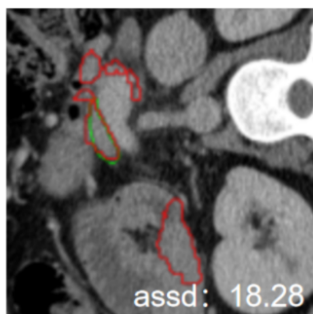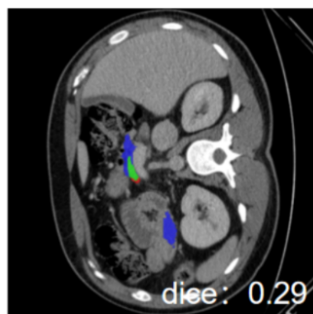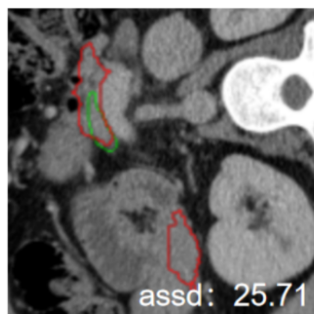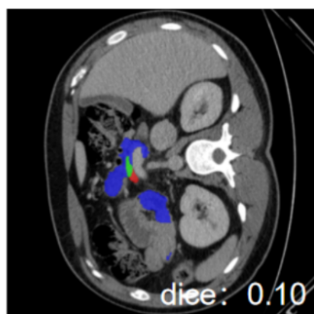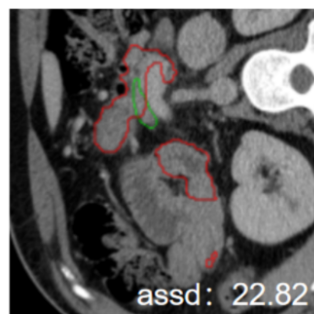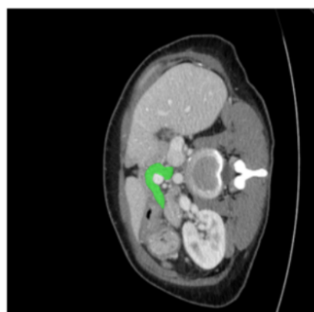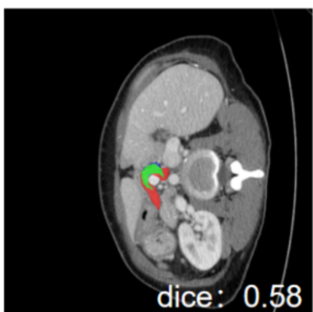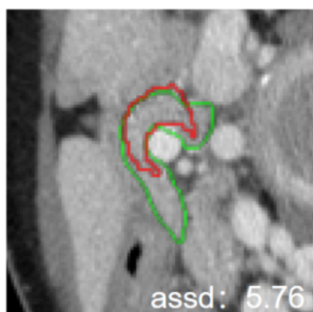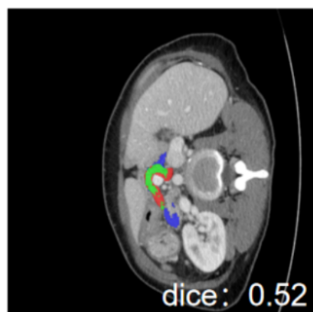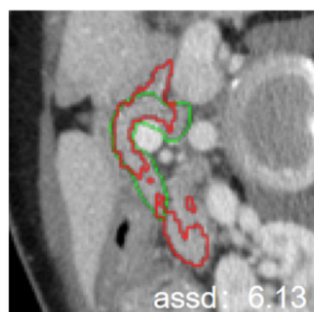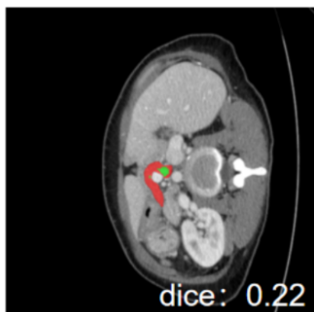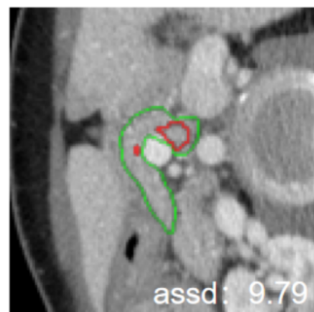

Supplement: S1 Fig — In columns 2, 4, and 6, the red regions represent FN (False Negative), the blue regions represent FP (False Positive), and the green regions represent TP (True Positive); in columns 3, 5, and 7, the red lines represent FN, and the green lines represent TP. (PDF) [file pone.0345549.s001.pdf]

(a) GroundTruth

(b) Ours

(c) ParaTransCNN

(d) TransUnet

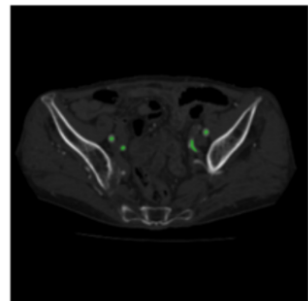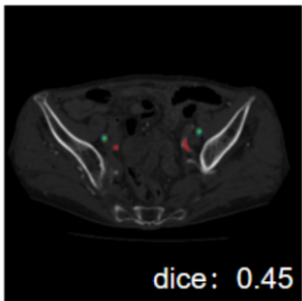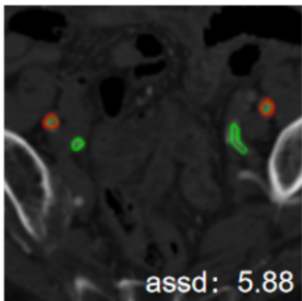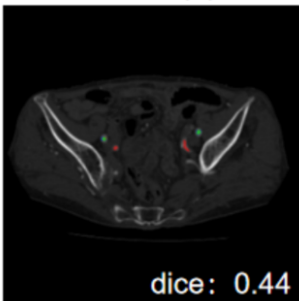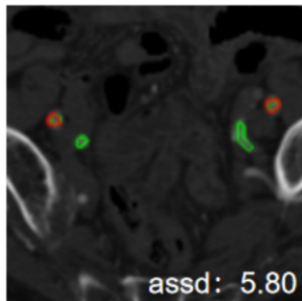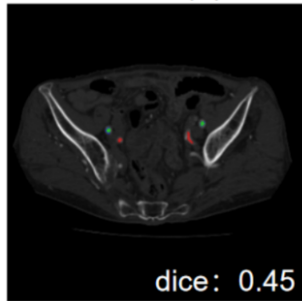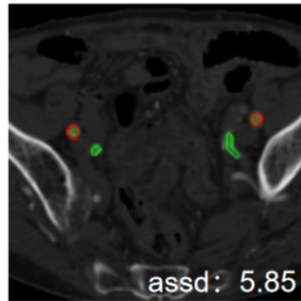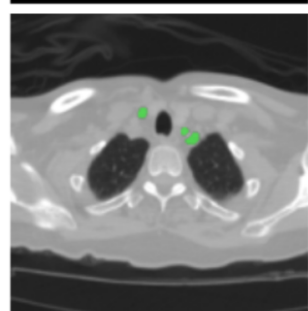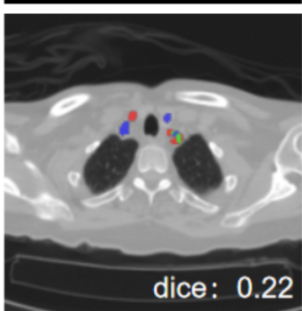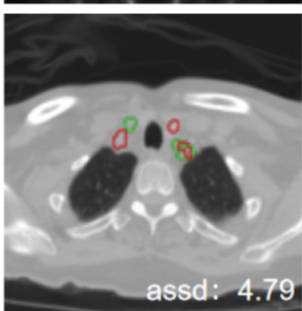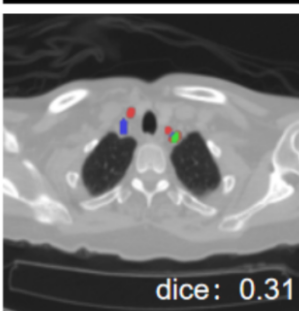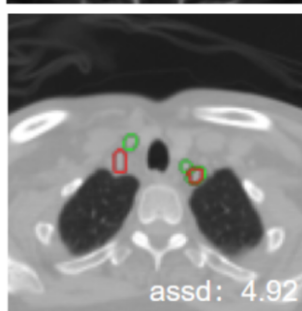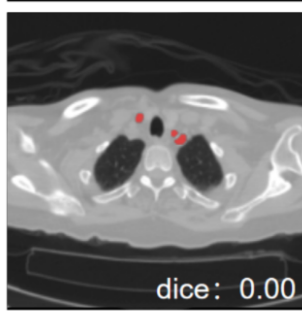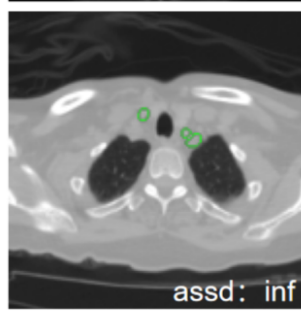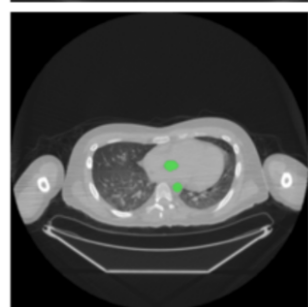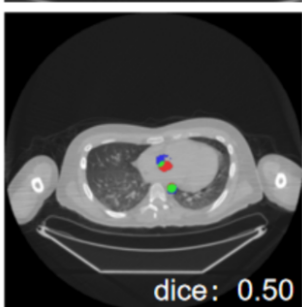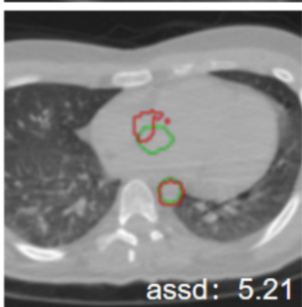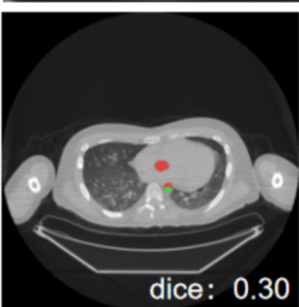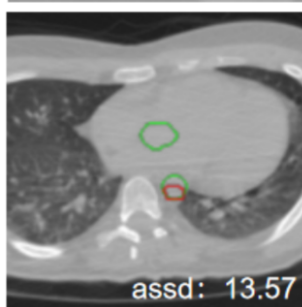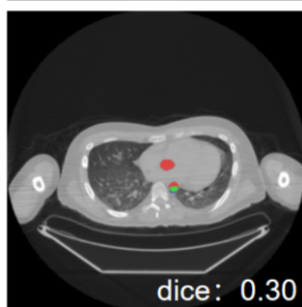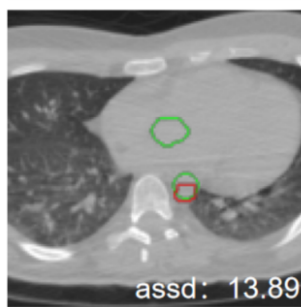

Supplement: S2 Fig — In columns 2, 4, and 6, the red regions represent FN (False Negative), the blue regions represent FP (False Positive), and the green regions represent TP (True Positive); in columns 3, 5, and 7, the red lines represent FN, and the green lines represent TP. (PDF) [file pone.0345549.s002.pdf]
