# Supplementary material for: A hybrid CNN-Transformer network integrating multiscale spatially detailed features for medical image segmentation
Source: PLoS One. 2026 Apr 29;21(4):e0345549. doi: 10.1371/journal.pone.0345549 (PMC13128111; doi:10.1371/journal.pone.0345549)
Supplement: S1 Table — (PDF) [file pone.0345549.s003.pdf]

S1 Table . Quantitative comparison of Dice score and HD95 on Synapse dataset across different random seeds.

Seed	Metric	Method	Avg	AO	GB	LK	RK	Liv	Pa	Sp	Sto
**1234**	**DSC (%)**	ParaTransCNN	83.49	88.61	72.26	87.97	82.83	94.82	68.59	90.55	82.32
		**Ours**	**84.64**	**89.25**	**73.16**	**88.22**	**85.11**	**95.26**	**71.60**	**91.78**	**82.73**
	**HD(mm)**	ParaTransCNN	16.06	13.10	**8.14**	35.38	10.96	11.05	9.00	32.15	**8.69**
		**Ours**	**12.96**	**10.89**	10.67	**24.42**	**10.46**	**6.72**	**8.01**	**16.20**	16.30
**6910**	**DSC (%)**	ParaTransCNN	82.63	88.57	67.18	87.20	82.09	94.76	**66.38**	**92.25**	**82.60**
		**Ours**	**83.34**	**89.13**	**69.30**	**89.15**	**85.82**	**95.42**	64.91	91.37	81.60
	**HD(mm)**	ParaTransCNN	18.11	11.92	14.10	33.55	39.37	14.22	10.93	**9.15**	**11.65**
		**Ours**	10.81	**5.35**	**7.20**	**3.84**	**15.47**	**8.57**	**10.19**	19.32	16.52
**7654**	**DSC (%)**	ParaTransCNN	79.85	88.12	64.58	82.79	73.02	94.66	65.17	**90.22**	80.23
		**Ours**	**84.29**	**89.85**	**76.74**	**88.05**	**84.64**	**95.41**	**70.04**	88.74	**80.88**
	**HD(mm)**	ParaTransCNN	28.33	8.24	29.25	49.59	61.90	**12.69**	10.65	43.55	**10.77**
		**Ours**	**12.50**	**7.93**	**17.49**	**43.80**	**12.04**	23.96	**9.58**	**31.93**	13.31
**9999**	**DSC (%)**	ParaTransCNN	81.94	87.88	69.05	84.09	78.96	94.65	66.64	90.35	**83.92**
		**Ours**	**84.33**	**88.93**	**72.36**	**88.34**	**85.76**	**95.17**	**69.45**	**91.88**	82.76
	**HD(mm)**	ParaTransCNN	19.86	9.04	**8.22**	37.33	55.86	19.81	10.59	**9.45**	**8.56**
		**Ours**	**11.30**	**4.78**	9.13	**3.08**	**8.30**	**13.01**	**7.01**	15.76	29.36
**5399**	**DSC (%)**	ParaTransCNN	81.26	88.45	64.64	84.52	80.10	94.98	64.23	91.90	81.23
		**Ours**	**84.33**	**89.71**	**74.20**	**88.24**	**84.96**	**95.21**	**68.95**	**92.04**	**81.34**
	**HD(mm)**	ParaTransCNN	22.08	11.25	23.14	54.39	32.47	**11.47**	12.61	20.38	**10.96**
		**Ours**	**15.61**	**4.75**	**19.70**	**26.65**	**19.82**	11.68	**8.73**	**19.33**	14.25

Note: Abbreviation: Avg, Average; AO, Aorta; GB, Gallbladder; LK, Left Kidney; RK, Right Kidney; Liv, Liver; Pa, Pancreas; Sp, Spleen; Sto, Stomach.
