# Supplementary material for: A hybrid CNN-Transformer network integrating multiscale spatially detailed features for medical image segmentation
Source: PLoS One. 2026 Apr 29;21(4):e0345549. doi: 10.1371/journal.pone.0345549 (PMC13128111; doi:10.1371/journal.pone.0345549)
Supplement: S2 Table — (PDF) [file pone.0345549.s004.pdf]

S2 Table . Quantitative results (Mean ± SD) and paired significance tests on the Synapse dataset.

Class	Metric	ParaTransCNN	Ours	p-value
**Avg**	**Dice** (%)	81.83 ± 1.39	$\mathbf{84.19 \pm 0.49}$	**0.016**
	**HD(mm)**	20.89 ± 4.70	$\mathbf{12.64 \pm 1.86}$	**0.008**
**AO**	**Dice(%)**	88.33 ± 0.31	$\mathbf{89.37 \pm 0.39}$	**0.007**
	**HD(mm)**	10.71 ± 2.01	$\mathbf{6.74 \pm 2.65}$	**0.021**
**GB**	**Dice(%)**	67.54 ± 3.23	$\mathbf{73.15 \pm 2.76}$	**0.002**
	**HD(mm)**	16.57 ± 9.61	$\mathbf{12.84 \pm 5.56}$	0.272
**LK**	**Dice(%)**	85.31 ± 2.19	$\mathbf{88.40 \pm 0.43}$	**0.026**
	**HD(mm)**	42.05 ± 9.33	$\mathbf{20.36 \pm 16.92}$	**0.016**
**RK**	**Dice(%)**	79.40 ± 3.89	$\mathbf{85.26 \pm 0.46}$	**0.025**
	**HD(mm)**	40.11 ± 19.95	$\mathbf{13.22 \pm 4.48}$	**0.042**
**Liv**	**Dice(%)**	94.77 ± 0.13	$\mathbf{95.29 \pm 0.11}$	**0.001**
	**HD(mm)**	13.85 ± 3.49	$\mathbf{12.79 \pm 6.75}$	0.803
**Pa**	**Dice(%)**	66.20 ± 1.63	$\mathbf{68.99 \pm 2.48}$	**0.038**
	**HD(mm)**	10.76 ± 1.29	$\mathbf{8.70 \pm 1.25}$	**0.029**
**Sp**	**Dice(%)**	91.05 ± 0.90	$\mathbf{91.16 \pm 1.39}$	0.865
	**HD(mm)**	22.94 ± 15.00	$\mathbf{20.51 \pm 6.60}$	0.707
**Sto**	**Dice(%)**	$\mathbf{82.08 \pm 1.37}$	81.86 ± 0.82	0.692
	**HD(mm)**	$\mathbf{10.13 \pm 1.37}$	17.95 ± 6.54	**0.049**

Note: Abbreviation: AO, Aorta; GB, Gallbladder; LK, Left Kidney; RK, Right Kidney; Liv, Liver; Pa, Pancreas; Sp, Spleen; Sto, Stomach.
