# Supplementary material for: A hybrid CNN-Transformer network integrating multiscale spatially detailed features for medical image segmentation
Source: PLoS One. 2026 Apr 29;21(4):e0345549. doi: 10.1371/journal.pone.0345549 (PMC13128111; doi:10.1371/journal.pone.0345549)
Supplement: S3 Table — (PDF) [file pone.0345549.s005.pdf]

S3 Table . Quantitative comparison of Dice score and HD95 on AVT dataset across different random seeds.

Seed	Method	All dataset		K dataset		R dataset		D dataset	
		DSC(%)	HD(mm)	DSC(%)	HD(mm)	DSC (%)	HD(mm)	DSC(%)	HD(mm)
**1234**	ParaTransCNN	87.27	**5.31**	**85.76**	7.32	82.06	**7.55**	93.99	1.07
	**Ours**	**88.24**	5.72	85.61	**4.92**	**85.12**	10.28	**94.09**	**1.06**
**6910**	ParaTransCNN	87.56	**4.86**	**85.82**	**5.83**	82.88	**7.69**	**93.99**	**1.07**
	**Ours**	**87.69**	5.94	85.62	5.92	**83.64**	10.77	93.83	1.14
**7654**	ParaTransCNN	87.70	**5.22**	**85.87**	6.84	83.38	**7.75**	**93.84**	**1.07**
	**Ours**	**87.91**	8.25	85.77	**6.78**	**84.25**	16.90	93.69	**1.07**
**9999**	ParaTransCNN	87.10	**5.33**	85.62	**5.92**	**83.64**	**10.76**	**93.83**	**1.13**
	**Ours**	**87.81**	8.54	**86.34**	7.87	83.30	16.62	93.80	1.14
**5399**	ParaTransCNN	87.89	**4.44**	85.67	**4.92**	**83.94**	**7.32**	**94.05**	1.07
	**Ours**	**87.91**	6.83	**86.18**	5.93	83.54	13.58	94.00	**1.00**
