# Supplementary material for: A hybrid CNN-Transformer network integrating multiscale spatially detailed features for medical image segmentation
Source: PLoS One. 2026 Apr 29;21(4):e0345549. doi: 10.1371/journal.pone.0345549 (PMC13128111; doi:10.1371/journal.pone.0345549)
Supplement: S4 Table — (PDF) [file pone.0345549.s006.pdf]

S4 Table . Quantitative results (Mean ± SD) and paired t-tests on the AVT dataset subsets across five random seeds.

Subset	Metric	ParaTransCNN	Ours	p-value
All dataset	Dice(%)	87.50 ± 0.32	$\mathbf{87.91 \pm 0.21}$	0.057
	HD(mm)	$\mathbf{5.03 \pm 0.38}$	7.06 ± 1.25	**0.047**
K dataset	Dice(%)	85.75 ± 0.10	$\mathbf{85.90 \pm 0.31}$	0.395
	HD(mm)	$\mathbf{6.17 \pm 1.05}$	6.28 ± 1.09	0.871
R dataset	Dice (%)	83.18 ± 0.74	$\mathbf{83.97 \pm 0.74}$	**0.043**
	HD(mm)	$\mathbf{8.21 \pm 1.44}$	13.63 ± 3.14	**0.003**
D dataset	Dice(%)	$\mathbf{93.94 \pm 0.08}$	93.88 ± 0.16	0.817
	HD(mm)	1.08 ± 0.03	$\mathbf{1.08 \pm 0.06}$	0.447
